# Linking organizational support with occupational satisfaction among Indian nurses

**DOI:** 10.6026/973206300214638

**Published:** 2025-12-15

**Authors:** Hepsi Bai Joseph, Vinitha Ravindran

**Affiliations:** 1 Department of Pediatric Nursing, College of Nursing, AIIMS, Bhubaneswar, Odisha, India; 2 Department of Pediatric Nursing, Former Dean, College of Nursing, Christian Medical College, Vellore, India

**Keywords:** Perceived organizational support (POS), occupational satisfaction, nurses, India, relationship

## Abstract

Organizational support is vital for enhancing job satisfaction among nurses, particularly in India, where workloads and limited resources are common. Therefore, it is of interest to examine the relationship between perceived organizational support (POS) and job satisfaction among 400 nurses in a tertiary care hospital in Kanyakumari, Tamil Nadu. Data collected using the Job Satisfaction Survey and POS Scale showed mean scores of 144 (±32) for satisfaction and 24 (±16) for POS. A significant positive correlation was found (ρ = 0.44; p <0.0001). Strengthening organizational support through recognition, benefits and fair policies can improve satisfaction, retention and care quality.

## Background:

In the context of healthcare, particularly among nurses, Perceived Organizational Support (POS) is crucial due to the high-stress environment and the demanding nature of the job. Job satisfaction among nurses is a multidimensional concept that encompasses satisfaction with various aspects of the job, including workload, work environment, relationships with colleagues, management practices and the alignment of personal and organizational values. Xu *et al.* emphasized the importance of organizational practices that foster a supportive work environment, such as recognizing and rewarding nurses' contributions, providing opportunities for professional growth and ensuring that nurses have the necessary resources to perform their duties effectively [1]. Another study by Karaszewski *et al.* similarly reported a positive correlation between POS and job satisfaction among hospital nurses in the United States, highlighting the critical role of organizational culture in shaping nurses' work experiences [2]. Li *et al.* emphasised POS and employee behavior, finding that POS is a strong predictor of job satisfaction and organizational commitment and that it can serve as a buffer against job stress and burnout [3]. Imran *et al.* found that POS is positively related to both organizational commitment and work engagement, which are key mediators in the relationship between POS and job satisfaction [4]. Gadolin *et al.* stated that the importance of POS is not only for nurses' job satisfaction but also for the overall quality of care provided in healthcare settings [5]. Zhou *et al.* emphasized the importance of organizational practices that support nurses' well-being, including providing access to mental health resources, promoting work-life balance and ensuring that nurses have the necessary tools and training to perform their duties effectively [6]. Therefore, it is of interest to link organizational support with occupational satisfaction among Indian nurses.

## Materials and Methods:

A correlational descriptive design was employed to investigate the objective of the present study among 400 nurses working in a selected tertiary care teaching hospital in Kanyakumari District, Tamil Nadu, India. Nurses were recruited using a simple random sampling method (random computer table) who were employed full-time in providing direct care to their patients, were able to read and write English/Tamil (the regional language) and were willing to participate in the study. Nurses who were working at a supervisory level were excluded. The sample size was calculated using a power analysis. Job satisfaction among nurses was assessed using the Job Satisfaction Survey (JSS), developed by Paul E. Spector, a standardized tool designed to measure employee attitudes towards job satisfaction [7]. It consists of 36 items, divided into nine subscales: Promotion, Pay, Supervision, Fringe Benefits, Contingent Rewards, Operating Procedures, Coworkers, Nature of Work and Communication. Each subscale is measured by four items, with some being reverse-scored to account for negatively worded statements. The tool uses a 6-point Likert scale, where responses range from "Disagree Very Much" (1) to "Agree Very Much" (6), providing a possible score range of 36 to 216. A higher score indicates greater job satisfaction. For assessing Perceived Organizational Support (POS) among nurses, the study employed the "Perceived Organizational Support Scale" developed by Eisenberger *et al.* [8]. This is a unidimensional scale that measures employees' perception of organizational support through eight items. It uses a 7-point Likert scale, where responses range from "Strongly Disagree" (0) to "Strongly Agree" (6), with reverse scoring applied to negatively worded items. The total score ranges from 0 to 48, where a higher score indicates a higher perceived level of organizational support. Study was informed to the nurses using a participant information sheet and informed consent was obtained from each participant, followed by administration of the self-report tool. Ethical permission was obtained from the Institutional Ethics Committee of Kanyakumari Medical College Hospital.

## Results and Discussion:

The demographic information revealed that the majority of nurses were between the ages of 31 and 35 and the majority of them were female (70%). Nurses represented the majority from wards (60%). The average monthly income was Rs. 25,000 and most of them worked in three shifts (68%), while the remaining worked on the general shift. Nurses reported an overall mean score of 144 (32) for job satisfaction and 24 (16) for perceived organizational support (Table 1 - see PDF). Nurses reported the highest mean scores in domains such as co-worker (20.9±4.5), nature of work (20.9±4.5), fringe benefits (18.4±6) and contingency reward (18.3±6) in job satisfaction domains (Table 2 - see PDF). There was a statistically significant correlation between perceived organizational support and job satisfaction among nurses (ρ = 0.44; p=<0.0001; 95% CI=0.359-0.517) (Table 3 - see PDF).

The overall mean score for job satisfaction among nurses in this study indicated moderate levels of job satisfaction. This finding is consistent with other recent studies. For instance, a study by Bhore' found that nurses' job satisfaction was moderately high, with organizational support, supervision and opportunities for promotion significantly influencing satisfaction levels [9]. Similarly, Gustavsson ' reported that job satisfaction among healthcare professionals is a critical factor in retention, burnout prevention and quality of patient care [10]. The perceived organizational support score in this study reflects moderate levels of organizational support. Research showed that perceived organizational support is a key determinant of job satisfaction, employee retention and psychological well-being [11]. In alignment with this, Galanis ' found that higher perceived organizational support was associated with better job satisfaction, reduced turnover intentions and improved performance among nurses [12]. Additionally, perceived organizational support has been linked to the perception of fairness, rewards and the quality of supervisor-subordinate relationships [13]. These findings suggest that enhancing organizational support, such as providing clear communication, recognition and professional development opportunities, can improve both job satisfaction and retention among nurses. The high score in the coworker domain aligns with studies showing that positive relationships with colleagues enhance job satisfaction. Zhang' found that strong coworker support fosters teamwork, reduces stress and increases job satisfaction among nurses. Furthermore, positive relationships among staff promote collaboration, which in turn improves patient care outcomes [14]. The high score in the nature of work domain reflects nurses' satisfaction with the meaningfulness and impact of their work, which is a well-documented contributor to job satisfaction. In a study by Dunlap' the nature of work was identified as one of the top factors associated with higher job satisfaction, as nurses often derive fulfilment from patient care and making a difference in patients' lives [15]. The scores for fringe benefits and contingent rewards highlight the importance of financial and non-financial incentives in promoting job satisfaction. Alahiane' emphasized that adequate benefits, including bonuses and recognition, enhance motivation and commitment in nursing staff [16]. Providing rewards based on performance is crucial for encouraging high-quality care and improving job retention rates. The significant correlation between perceived organizational support (POS) and job satisfaction among nurses in this study is consistent with existing research highlighting the strong link between organizational support and employee satisfaction which suggests that as nurses perceive greater organizational support, their job satisfaction increases. This finding aligns with the study by Sharif' which found that employees who feel supported by their organization report higher job satisfaction and lower turnover intentions [17]. Organizational support, including resources, recognition and fair treatment, fosters a sense of belonging and value, which in turn boosts morale and job satisfaction [18]. Additionally, studies by Markovic ' have demonstrated that POS not only influences job satisfaction but also mitigates burnout and stress among healthcare workers, highlighting its protective role in high-stress environments like nursing [19]. Enhancing perceived organizational support through leadership engagement, professional development opportunities and recognition programs can, therefore, have a significant impact on nurse satisfaction and retention. As the results of this study show, improving POS could be an effective strategy to increase job satisfaction and overall well-being among nursing professionals. Improving perceived organizational support (POS) and job satisfaction among nurses in India requires a multifaceted approach, involving both individual and systemic efforts. Nurses can play an active role in enhancing their work environments, but organizational and policy-level changes are equally essential. One of the most effective ways nurses can improve POS is by fostering positive relationships with their supervisors and peers. Effective communication with leadership is crucial for receiving adequate support and the necessary resources. Studies have shown that open and transparent communication between staff and management can significantly improve perceptions of organizational support [20]. Nurses should also seek regular feedback from their supervisors; express their concerns and work collaboratively to create a supportive work atmosphere. A key factor in both job satisfaction and POS is the presence of a supportive peer network. Nurses should actively engage in teamwork and offer emotional and professional support to their colleagues. In a study by Norling ' coworker support was shown to reduce stress and burnout while enhancing job satisfaction [21]. This is particularly important in high-stress environments, such as Indian healthcare, where nurses often face heavy workloads and emotional strain. Establishing a culture of mutual support can foster a more positive work environment and improve overall satisfaction. Continual professional development is another way nurses can enhance both patient outcomes and job satisfaction. By actively seeking opportunities for in-service training, workshops and certifications, nurses can demonstrate their commitment to personal growth and professional excellence. Participation in continuing education not only enhances job performance but also helps nurses feel more valued by their organizations, thus improving POS [22]. Hospitals and nursing institutions in India should create structured professional development programs to support nurses in their career advancement. Nurses should advocate for better recognition and reward systems, including both financial and non-financial incentives. Recognition through promotions, bonuses and other performance-based rewards can significantly enhance job satisfaction [16]. Nurses can work collectively, through professional associations and unions, to push for better pay scales, improved working conditions and fair staffing ratios. This is especially relevant in the Indian context, where many nurses feel underpaid and undervalued despite their critical role in the healthcare system. Involving nurses in organizational decision-making processes can significantly boost their sense of organizational support. When nurses are included in discussions about policies and procedures that affect their work, they feel more valued and engaged. Research has shown that participatory decision-making leads to higher job satisfaction and retention [23]. In India, where hierarchical structures often dominate, creating more opportunities for nurses to voice their opinions can lead to greater empowerment and a stronger sense of organizational commitment.

## Strengths and limitations:

The study is conducted in an Indian setting, making the findings highly relevant to the specific challenges faced by nurses in Indian healthcare systems and those from developing countries. This helps address the unique socio-cultural, economic and organizational factors that influence job satisfaction and perceived organizational support (POS) in India. The study employed widely recognized and validated tools, such as the Job Satisfaction Survey (JSS) and the Perceived Organizational Support Scale (POS), which ensures the reliability and validity of the findings. The use of statistical correlation between POS and job satisfaction adds scientific rigor to the findings, providing concrete evidence of the relationship between organizational support and job satisfaction in the Indian nursing context. The results provide practical insights for healthcare administrators and policymakers in India, highlighting the importance of effective organizational support systems in enhancing job satisfaction, reducing burnout and improving retention rates among nurses. The study has limitations, as it was conducted in a specific geographical region and healthcare institution, which limits its generalizability to the broader nursing population across India, particularly in rural and underserved areas. Longitudinal studies would provide a more dynamic understanding of these variables.

## Conclusion:

Perceived organizational support (POS) strongly influences job satisfaction among nurses in India, where high workloads, limited resources and inadequate staffing increase workplace challenges. Enhancing organizational support through recognition, peer support, professional development and participatory decision-making can significantly improve POS and job satisfaction, strengthen retention and reducing burnout. Adopting international best practices such as structured mentorship, better staffing ratios and clear career pathways can further elevate nurse satisfaction and care quality, benefiting healthcare systems in India and globally.
